# Predicted Weight of Infants According to Biological, Psychological and Social Variables of Mothers in Iran

**Published:** 2017-04

**Authors:** Mohamad ZARENEYESTANAK, Masoud Gholamali LAVASANI, Gholamali AFROOZ

**Affiliations:** 1. Dept. of Psychology and Education of Exceptional Children, Science and Research Branch, Islamic Azad University, Tehran, Iran; 2. Dept. of Psychology and Educational Sciences, School of Psychology, University of Tehran, Tehran, Iran

## Dear Editor-in-Chief

One of the most serious developmental problems in children is low birth weight in the world today (1). Babies born weighing less than 2500 gr are considered as LBW infants during birth (2). Evaluation that as the cause of defects of interest is biological and emotional. At least 18 million children are born underweight, contains with about 14% of births (3). Therefore, one of the main cause’s prevention strategies of disability is providing measures to identify the possible causes of this problem and to know statistics how reduce LBW infants (4). To achieve, all factors must associate with low birth weight, especially biological and psychosocial characteristics of parents recognized the results of the preventive measures taken. The mortality rate in Iran for children under 5 yr, is about 32 cases per thousand and children under 2 yr, is 26 per thousand that 18 per thousand of them die in the first month and most of them are infants with low birth weight. The rate of Low weight of birth has been reported 8% in Iran (5). Several factors such as low socio-economic level, poor nutrition, anemia, different diseases, lack of access to health care during pregnancy, obstetric complications, abortion, pregnancy at an early age, pregnancy in low distance, low height and weight and drug use are associated with LBW(6).

A high prevalence of psychiatric disorders was showed such as anxiety and depression during pregnancy and poor in middle-income countries (7). Consequences of mental health problems of mothers will be infants with reduced motor development, behavioral changes, digestive problems and developmental delay. The mental state of the mother during pregnancy affects fetal health (8). However, Iran has been little research in this area, the point of this research is investigation of the biological, cognitive and environmental characters in mother’s underweight and optimal weight infants. The research methods causal-comparative was conducted.

The study population included all infants born LBW and weight between the end of 2012–2013 in Isfahan Province with their parents. LBW rates in the Isfahan Province was reported about 10%, therefore, the total sample consisted of 400 live births in the period mentioned. Of these, 200 babies with low birth weights and weight are desirable. Stratified according to the population in each city was conducted. In order to gather information on the properties of biological and environmental parental questionnaire for the first time (1), was set to be used, also the DAS scales of depression, anxiety, and stress were used to collect the data.

Reliability of the questionnaire through Cronbach’s alpha obtained varies from 0.85 (9). A stepwise regression method is used to express the impact of these variables (independent) variables in predicting birth weight (dependent variable). Pearson correlation coefficients double-stranded were used for this purpose, and according to the available information based on the scale is ordinal and interval.

According to Table 1, the coefficients of the entered variables in the regression model are significant systematically. Means for the preterm types, anxiety, distance, depression, euphoria, hypertension, and birth order regression model showed that in the response variables that include impact on infants’ weight there is a significant relationship (*P*<0.05).

**Table 1:** Regression coefficients with significance test and separated regression coefficients

**Model**	**B**	**S.E**	**β**	**T**	**Sig**	**r**	**Part**	**Partial**
Premature	−889.218	55.461	−0.549	−16.033	0.001	−0.742	−0.629	−0.470
Anxiety	−42.179	−9.279	−0.185	−4.545	0.001	−0.508	−0.224	−0.133
Interval	44.122	12.996	0.108	3.395	0.001	0.397	0.169	0.100
Depression	−43.555	8.558	−0.269	−5.089	0.001	−0.504	−0.249	−0.149
Satisfaction	−3.685	1.003	−0.170	−3.675	0.001	0.228	−0.182	−0.108
Blood pressure	−275.669	89.04	−0.092	−3.107	0.001	−0.138	−0.155	−0.091
Order	46.889	17.042	0.083	2.751	0.002	0.228	0.138	0.081

Mental, physical and emotional state of mother can affect the incidence of LBW. Finally, with increasing the quality of life and prevention of other risk factors, LBW can decrease.

## References

[1] Afrouz GH Parand A (2008). Analyze the relationship between birth weight and prevalence of biological damage, cognitive and motor. Journal of Psychology and Educational Sciences, 37(2):123–131(in Persian).

[2] Akin Y Comert S Turan C Unal O Picak A Ger L Telatar B (2010). Increasing low birth weight rates: deliveries in a tertiary hospital in Istanbul. Iran J Pediatr, 20(3):284–290. 23056718 PMC3446039

[3] Rondo PH (2007). Maternal stress\distress and low birth weight, preterm birth and intrauterine growth restriction- A review. Current Women ‘S Health Reviews, 3(1): 13–29.

[4] Abasalti Z Abrishami M Nazeranpour F (2006). The prevalence of low birth weight in the population covered by the University in Khorasan province. Iranian Nutrition Congress, Iran.

[5] Poyner Del Vento PW Cobb RJ (2011). Chronic Stress as a moderator of the association between depressive symptoms and marital satisfaction. J Clin Psychol, 30(9): 905–936.

[6] Eghbalian F (2008). Factors associated with low birth weight. Iran J Pediatr, (17):27–33 (in Persian).

[7] Murphy CC Schei B Myhr Tl (2001). Abuse: a risk factor for low Birth weight? A systematic review and meta-analysis. CMAJ, 164(11):1567–72. 11402794 PMC81110

[8] Kay RE (2009). Maternal Stress and Infant Outcomes: The Impact of Perinatal Anxiety on Pregnancy and Delivery Outcomes [PhD thesis]. University of Michigan, USA.

[9] Silva R Thomas M Caetano R Aragaki C (2006). Preventing low birth weight in Illinois: Outcomes of the family case management program. Matern Child Health J, 10(6): 481–488. 16865536 10.1007/s10995-006-0133-8

